# Bedarfe der Langzeitpflege in der COVID-19-Pandemie

**DOI:** 10.1007/s00391-020-01801-7

**Published:** 2020-10-28

**Authors:** Claudia Stolle, Annika Schmidt, Dominik Domhoff, Anna Carina Friedrich, Franziska Heinze, Benedikt Preuß, Kathrin Seibert, Heinz Rothgang, Karin Wolf-Ostermann

**Affiliations:** 1 Institut für Public Health und Pflegeforschung (IPP), Universität Bremen, Bremen, Deutschland; 2 SOCIUM, Universität Bremen, Bremen, Deutschland; 3 Hochschule Bremen, Neustadtswall 30, 28199 Bremen, Deutschland

**Keywords:** SARS-CoV‑2, Corona, Altenpflege, Ambulante Pflege, Wünsche, SARS-CoV‑2, Corona, Eldery care, Outpatient nursing, Wishes

## Abstract

Das Virus SARS-CoV‑2 und die damit einhergehende Erkrankung COVID-19 stellen die Gesundheitssysteme weltweit vor große Herausforderungen. Besonders die vulnerable Gruppe pflegebedürftiger Menschen in der Langzeitpflege ist gefährdet, schwere Krankheitsverläufe zu erleiden oder an der Infektion zu versterben.

In einer bundesweiten Querschnittsstudie wurden mittels einer Onlinebefragung die Situation und die Bedarfe stationärer und ambulanter Langzeitpflegeeinrichtungen während der SARS-CoV-2-Pandemie erfasst und analysiert.

Konkret forderten Teilnehmende aus 531 Einrichtungen einheitliche Handlungsempfehlungen zu SARS-CoV‑2, ausreichende und finanzierbare Schutz- und Hygienematerialien, Reihentestungen in den Einrichtungen, fundierte Beratung bei der Umsetzung von Maßnahmen, einen konkreten Pandemieplan und unterstützende Öffentlichkeitsarbeit in den Medien. Dazu wurden eine höhere Vergütung der Pflegenden, eine bessere Personalausstattung und stärkere Wertschätzung der pflegerischen Profession gefordert.

Um die vulnerable Gruppe pflegebedürftiger Menschen vor einer SARS-CoV-2-Infektion zu schützen, muss die Langzeitpflege stärker in den Fokus der gesundheitspolitischen Maßnahmen während der Pandemie gerückt werden.

## Abstract (en)

The SARS-CoV‑2 virus and the associated disease COVID-19 pose major challenges to healthcare systems worldwide. Especially the vulnerable group of people in need of long-term care is at risk of suffering a severe course of the disease or of dying from the infection.

In a nationwide cross-sectional study the situation and needs of inpatient and outpatient long-term care facilities during the SARS-CoV‑2 pandemic were assessed and analyzed using an online survey.

Participants from 531 institutions postulated the need for uniform recommendations for action on SARS-CoV‑2, adequate and affordable protective and hygiene materials, serial tests in the institutions, well-founded advice on the implementation of interventions, a specific pandemic plan and supporting public relations work by the media. This calls for higher nursing remuneration, better staffing levels and greater appreciation of the nursing profession.

In order to protect the vulnerable group of people in need of nursing care from a SARS-CoV‑2 infection, long-term care must be given a stronger focus in health policy measures during the pandemic.

## Hinführung zum Thema

Das „severe acute respiratory syndrome coronavirus type 2“ (SARS-CoV-2) trat erstmalig im Dezember 2019 in der chinesischen Stadt Wuhan auf [10]. Eine Ansteckung erfolgt hauptsächlich durch die respiratorische Aufnahme viraler Aerosole, die z. B. beim Husten, Niesen, Atmen oder Sprechen freigesetzt werden. Zu den häufigsten Symptomen gehören Husten, Fieber, Schnupfen sowie Geruchs- und Geschmacksverlust. Der Krankheitsverlauf variiert von symptomlosen Fällen bis zu schweren Lungenentzündungen mit Todesfolge [8, 9]. Die rapide Ausbreitung des SARS-CoV‑2 und die damit einhergehenden Erkrankungsfälle an COVID-19 (coronavirus disease 2019) stellen die Gesundheitssysteme weltweit vor immense Herausforderungen. Der Fokus der medialen Berichterstattung lag bisher primär auf der Verhinderung einer Ausbreitung des Virus in der Bevölkerung und in der Betrachtung der Akutversorgung Erkrankter in Krankenhäusern. Ein weiterer zentraler Ort des Infektionsgeschehens sind stationäre und ambulante Pflegeeinrichtungen. Pflegebedürftige Menschen bilden hier mit einer Fallsterblichkeit von 19 % bei einer SARS-CoV-2-Infektion eine wesentliche Risikogruppe [4]. Dazu haben im Vergleich zur Gesamtbevölkerung Pflegende in der stationären Langzeitpflege ein 6‑faches und in der ambulanten Versorgung ein 3‑faches Risiko, an COVID-19 zu erkranken [15]. In den Medien wurden v. a. Besuchsverbote in Pflegeeinrichtungen und die Auswirkungen auf die Pflegebedürftigen und deren Angehörigen thematisiert. Unklar bleibt bislang jedoch, welche Ressourcen und unterstützende Maßnahmen Pflegeeinrichtungen während der COVID-19-Pandemie benötigen, um eine qualitativ hochwertige pflegerische Versorgung der Pflegebedürftigen sicherzustellen.

## Hintergrund

Die COVID-19-Pandemie fordert die Gesundheitssysteme weltweit in bisher nicht gekanntem Ausmaß. Bis zum 01.10.2020 wurden in Deutschland ca. 290 Tsd. laborbestätigte SARS-CoV-2-Fälle sowie rund 9500 Todesfälle im Zusammenhang mit COVID-19 an das Robert Koch-Institut (RKI) übermittelt [7]. Pflegebedürftige Menschen gehören zu den vulnerablen Gruppen, die bei einer Infektion in besonderem Maße von schweren Krankheitsverläufen und einer hohen Sterblichkeit betroffen sein können [4, 9]. Nach aktuellen Hochrechnungen sind knapp 50 % der an COVID-19 Verstorbenen in Deutschland Bewohnerinnen und Bewohner von Pflegeheimen gewesen [11]. Treten SARS-CoV-2-Infektionen in Pflegeeinrichtungen auf, kommt es mit durchschnittlich 18,8 Fällen pro Einrichtung zu einem hohen Infektionsgeschehen [4]. In anderen europäischen Ländern (Italien, Spanien, Frankreich, Irland oder Belgien) stellen Verstorbene in Pflegeheimen ebenfalls etwa die Hälfte der Todesfälle mit COVID-19 dar [3, 4]. Vor diesem Hintergrund erscheint der Schutz von in einem Pflegheim lebenden Menschen vor einer SARS-CoV-2-Infektion von besonderer Bedeutung. Angesichts einer sowohl räumlich nahen Unterbringung als auch der Erbringung körperlich naher pflegerischen Tätigkeiten ergibt sich hier eine neuartige Herausforderung für Pflegeeinrichtungen. In diesen leisten v. a. Pflegende durch die Einhaltung und Gewährleistung von Schutzmaßnahmen einen essenziellen Beitrag zum Schutz der Pflegebedürftigen vor einer SARS-CoV-2-Infektion. Allerdings war die Personalsituation in stationären Pflegeeinrichtungen bereits vor der COVID-19-Pandemie angespannt. So wurde im Jahr 2019 im Mittel ein Personalmehrbedarf von mehr als einem Drittel pro stationärer Pflegeeinrichtung ermittelt [12, 13]. Studien berichten, dass dieser Personalmangel durch die Folgen des Ausbruchs der Pandemie weiterverschärft wurde [11, 15]. Pflegerisches Personal ist im Vergleich zu nichtmedizinischem Personal vermehrt von einer SARS-CoV-2-Infektion betroffen [11] und fällt in Verdachtsfällen durch das Warten auf das Vorliegen eines Testergebnisses und eine Selbstisolation häufiger als Arbeitskraft aus. Gleichzeitig entsteht durch die Umsetzung erweiterter Hygienemaßnahmen ein deutlich erhöhter pflegerischer Arbeitsmehraufwand [11, 15]. Um eine fachgerechte Versorgung der Pflegebedürftigen weiterzusichern, müssen die beschriebenen Personalausfälle und Arbeitsmehraufwände durch die Pflegeeinrichtungen kompensiert werden [11, 15]. Kenntnisse über die Unterstützungsbedarfe der Pflegepraxis während der COVID-19-Pandemie tragen dazu bei, auf gesundheitspolitischer Ebene spezifische Unterstützungsangebote initiieren zu können.

## Ziele der Arbeit und Fragestellung

Ziel dieser Arbeit ist es, die Erfahrungen der Pflegeeinrichtungen aus der Zeit der abgeklungenen ersten Welle der COVID-19-Pandemie in Deutschland zu nutzen und daraus weitreichende Lehren zu ziehen. Leistungserbringer, aber auch politische Akteure können die Erkenntnisse dieser Arbeit aufgreifen, um den zukünftigen Herausforderungen der nunmehr wieder ansteigenden Infektionszahlen besser zu begegnen und Pflegeeinrichtungen eine gezielte Unterstützung etwa zur Kompensation der Mehraufwände und der arbeitsorganisatorischen Herausforderungen durch die COVID-19-Pandemie anzubieten.

Vor diesem Hintergrund führte die Universität Bremen eine Untersuchung zur Situation der Langzeitpflege während der COVID-19-Pandemie durch. In Ergänzung zu weiteren Studienergebnissen [11, 15] fokussiert dieser Artikel auf die Fragestellung: Welche unterstützenden Maßnahmen erachten die Einrichtungen während der Pandemie als sinnvoll, um eine qualitativ hochwertige Versorgung der Pflegebedürftigen sicherzustellen?

Aus den durch die Einrichtungen benannten Bedarfen sollen gezielte Maßnahmen abgeleitet werden, die während der COVID-19-Pandemie sowohl das Risiko für das Auftreten eines akuten Infektionsgeschehens mindern helfen als auch im Umgang mit einem solchen unterstützen können.

## Studiendesign und Untersuchungsmethoden

### Studiendesign

Bei der vorliegenden Studie handelt es sich um eine bundesweit durchgeführte Onlinebefragung im Querschnittsdesign. Hierzu wurde ein Fragebogen entwickelt und per E‑Mail ein Befragungslink an eine Gelegenheitsstichprobe von 7723 Pflegeheimen (53 % aller Pflegeheime in Deutschland [14]), 9547 ambulanten Pflegediensten (68 % aller Pflegedienste in Deutschland [14]) und 2731 teilstationären Einrichtungen versandt. Die Kontaktadressen der Einrichtungen wurden aus den öffentlich zugänglichen Profilen des AOK-Pflege-Navigators entnommen [1]. Darüber hinaus informierten Trägerverbände ihre Mitglieder über die Befragung und luden diese zur Teilnahme ein. Die Befragung fand vom 28.04.2020 bis zum 12.05.2020 statt. Je nach Fragestellung bezog sich der Auskunftszeitraum für die Befragten auf den gesamten Zeitraum der Pandemie bis zum Tag der Fragebogenbearbeitung oder z. B. zur Berechnung von Personalausfällen auf ein eng definiertes Zeitintervall. Neben Fragen zu Strukturmerkmalen der Einrichtungen beinhaltet der Fragebogen u. a. Fragenkomplexe zum Vorkommen des SARS-CoV‑2 in den Einrichtungen und zu Folgen und Auswirkungen der Pandemie etwa auf die Ausstattung, die Arbeitsprozesse und Kommunikationsstrukturen [11, 15]. Die Einrichtungen erhielten weiter die Möglichkeit, aus ihrer Praxisperspektive heraus konkrete Maßnahmen zu formulieren, die sie während der Pandemie zur Unterstützung der Sicherstellung der Versorgung als sinnvoll erachtet hätten. Insgesamt nutzten 531 Befragte diese offene Antwortmöglichkeit (286 Befragte der stationären Langzeitpflege, 209 Befragte aus ambulanten Pflegediensten, 29 aus dem Bereich der teilstationären Pflege und 7 Personen aus anderen Einrichtungsformen).

### Datenaufbereitung und -auswertung

Die Antworten in den Freitextfeldern wurden in die qualitative Datenauswertungssoftware f4analyse 2.4.2 EDUCATION (audiotranskription, Marburg, Deutschland) eingelesen, inhaltsanalytisch gebündelt und in ein Kategoriensystem überführt. Die Auswertung erfolgte dabei in Anlehnung an Kuckartz 2018 [5] in 7 Phasen: 1. initiierende Textarbeit, Markieren und Codieren wichtiger Textstellen, Schreiben von Memos, 2. über ein induktives Vorgehen die Anlegung eines vorläufigen Kategoriensystems mit Ober‑, Unter- und Subkategorien, 3. in einem ersten Codierprozess, Codieren des Materials in die bestehenden Kategorien, 4. Zusammenstellen aller Textstellen mit gleicher Kategorie, 5. induktives Ausdifferenzieren von Subkategorien im Material, 6. zweiter Codierprozess, Codieren des Materials in das ausdifferenzierte Kategoriensystem, 7. Kontrolle und Überarbeitung der Zuordnung des Materials im entwickelten Kategoriensystem.

## Ergebnisse

### Beschreibung der Stichprobe

Aus allen Bundesländern nahmen insgesamt 823 Pflegeheime 10,7 % der angeschriebenen Heime an der Befragung teil. Zudem nahmen insgesamt 701 ambulante Pflegeeinrichtungen (Pflegedienste, Rücklauf 7,3 %) an der Befragung teil (Tab. 1). Die Fragebogen wurden von Einrichtungsleitungen, Pflegedienstleitungen oder Qualitätsbeauftragten ausgefüllt. 531 Befragte nutzten die Möglichkeit, in den Freitextangaben ihre Bedarfe während der COVD-19-Pandemie zu benennen, was an dieser Stelle die Bedeutung des Themas für die Pflegeeinrichtungen verdeutlicht.

**Table 1:** 

Merkmal	Stationär		Ambulant	
	Stichprobe (*n* = 824)	Pflegestatistik (*n* = 764.648)	Stichprobe (*n* = 701)	Pflegestatistik (*n* = 390.322)
*Einrichtungsgröße (Mittelwert)*				
Zahl der Plätze	90,6 (782)	78,0	–	–
Bewohner*innen	83,7 (774)	70,5		
*Mitarbeitende (Mittelwert)*				
Personenzahl	87,0 (774)	52,8	37,5	27,8
Vollzeitäquivalente	50,7 (707)	38,2	19,9	18,9
*Pflegekräfte (Mittelwert)*				
Personenzahl	50,8 (734)	38,4	25,8	20,1
Vollzeitäquivalente	33,0 (687)	28,9	15,6	13,9
*Trägerschaft*				
Öffentlich	11,0 % (89)	5,3 %	5,0 %	1,4 %
Freigemeinnützig	53,4 % (431)	53,6 %	33,8 %	32,8 %
Privat	35,6 % (287)	41,1 %	61,1 %	65,8 %

Vergleichsquellen der amtlichen Statistik: Statistisches Bundesamt, 2019

Hinsichtlich der Strukturmerkmale sind die teilnehmenden Einrichtungen weitestgehend mit den Ergebnissen der Pflegestatistik 2017 [14] vergleichbar, jedoch sind auch Abweichungen festzustellen (Tab. 1).

### Auswertung der Freitextangaben

**Figure 1:**
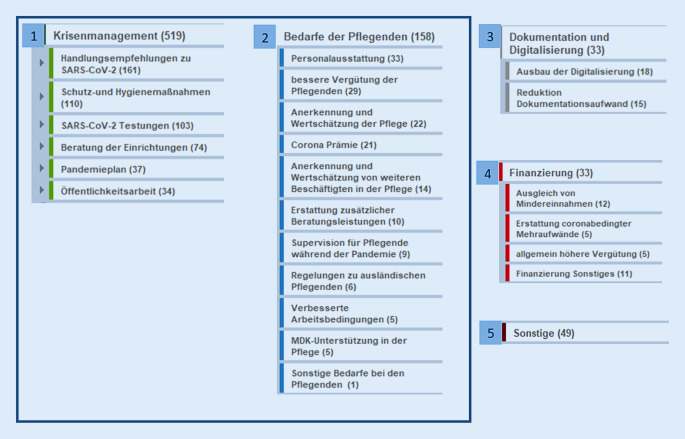

Die Textstellen der Freitextangaben ließen sich 5 Kategorien zuordnen: Krisenmanagement (519 Textstellen), Bedarfe der Pflegenden (158 Textstellen), Dokumentation und Digitalisierung (33 Textstellen), Finanzierung (33 Textstellen) und Sonstiges (49 Textstellen) (Abb. 1).

Dieser Artikel berichtet die Aussagen zu den 2 Oberkategorien mit den häufigsten zugeordneten Textstellen: Krisenmanagement und Bedarfe der Pflegenden. Aussagen zu den Kategorien Dokumentation und Digitalisierung, Finanzierung und sonstige Bedarfe (Abb. 1) werden an dieser Stelle aus Platzgründen nicht berichtet. Aufgrund der inhaltlichen Nähe und fehlender Trennschärfe der Freitextangaben zwischen den befragten Versorgungssettings wurde in der Auswertung der Freitexte keine Unterscheidung nach Versorgungsformen vorgenommen. Die berichteten Ergebnisse beziehen sich daher sowohl auf die stationäre als auch auf die ambulante Langzeitpflege.

Die in Klammern aufgeführten Zahlen geben die Anzahl der zugeordneten Textstellen an. Da eine Textstelle auch mehreren Kategorien zugeordnet werden konnte, können Abweichungen zur Anzahl der Befragten vorliegen.

### Bedarfe im Bereich Krisenmanagement

Der Oberkategorie Krisenmanagement ließen sich die meisten Textstellen (*n* = 519) zuordnen. In der hier subsumierten Unterkategorie *Handlungsempfehlungen zu SARS-CoV‑2* formulierten 43,5 % der Befragten (*n* = 70 Textstellen) einen deutlichen Bedarf an (bundes‑)*„einheitlichen Empfehlungen“ *(Abb. 1). Die Teilnehmenden sahen sich bei der Umsetzung der offiziellen Vorgaben teilweise *„widersprüchlichen Angaben“ *seitens des RKI oder der Gesundheitsämter und Träger ausgesetzt. In der Konsequenz wurden von den Befragten *„einheitliche“ *sowie *„koordinierte Vorgaben“ *und Empfehlungen verlangt.

Ebenfalls häufig, von rund 15 % der Befragten, wurde eine *„schlechte Umsetzbarkeit“* der Empfehlungen und Vorgaben (*n* = 24) genannt. Hier wurde gefordert, Empfehlungen und Regelungen im Zusammenhang mit der Pandemie *„praxisnah“* zu erarbeiten und Pflegepraxis sowie Pflegewissenschaft in die Entwicklung miteinzubinden, sodass Vorgaben entstünden, die für die Praxis auch anwendbar seien. Des Weiteren wurden *„verständliche“ *und *„klare Informationen“* (*n* = 22) sowie beständige und *„verlässliche“* Empfehlungen (*n* = 18) gefordert, die nach aufwendiger Umsetzung längere Gültigkeit hätten. Zur Umsetzung von Vorgaben (*n* = 17) benötigten die Einrichtungen *„ausreichend Zeit*“. Zehn Textstellen bezogen sich auf einen Bedarf an demenzspezifischen Regelungen. Zum Zeitpunkt der Befragung lägen den Einrichtungen keine Aussagen zum spezifischen Umgang mit demenziell Erkrankten vor, etwa in Bezug auf das Umherwandern von Menschen mit Demenz oder deren fehlendes Verständnis von Präventionsmaßnahmen, vor. Hier wiesen die Befragten auf eine Lücke bei notwendigen Informationen und Verfahrenshinweisen im Umgang mit kognitiv beeinträchtigten und verhaltensveränderten Pflegebedürftigen während der COVID-19-Pandemie hin.

Die Unterkategorie *Schutz- und Hygienemaßnahmen* beinhaltete Forderungen nach *„ausreichender Bereitstellung von Schutzkleidung“ *und *„Desinfektionsmitteln“* (*n* = 46) sowie einer besseren Organisation der Schutz- und Hygienematerialien (*n* = 35). Die Beschaffung von Sachmitteln (Schutzausrüstung etc.) wurde von den Befragten als sehr aufwendig beschrieben und der Aufbau zentraler Lager und Verteilsysteme für die Einrichtungen im Gesundheitswesen vorgeschlagen. In diesem Zusammenhang wurde auch eine *Preiskontrolle* der Schutzmaterialien (*n* = 20) durch die Befragten benannt. Einige Teilnehmende gaben an, während der Pandemie „*überhöhte Preise*“ für Waren gezahlt zu haben, was langfristig nicht tragbar sei.

70 % der Befragten, zusammengefasst in der *Unterkategorie *SARS-CoV-2-*Testungen*, forderten die Durchführung von *„systematischen“ *und *„regelmäßigen“* Reihentestungen (*n* = 72) zum einen beim Personal der Einrichtungen und zum anderen bei den Pflegebedürftigen. Die Testungen seien notwendig, um schnell Infektionsherde zu erkennen, in positiv getesteten Fällen rasch handeln zu können und eine weitere Verbreitung des SARS-CoV‑2 zu verhindern. Dazu wurde auf die Notwendigkeit *„schnellerer Testungen und Testergebnisse*“ (*n* = 17) hingewiesen, damit Pflegende mit negativem SARS-CoV-2-Test kurzfristig wieder den Dienst aufnehmen können. Weiter wurde eine *„bessere Kommunikation“* der Testergebnisse (*n* = 8) gewünscht, so wurden diese beispielsweise nicht an Pflegeeinrichtungen übermittelt, sodass bei Neuaufnahmen oder Überleitungen von Pflegebedürftigen den Einrichtungen nicht bekannt war, ob bei diesen eine Infektion vorlag.

**Figure 2:**
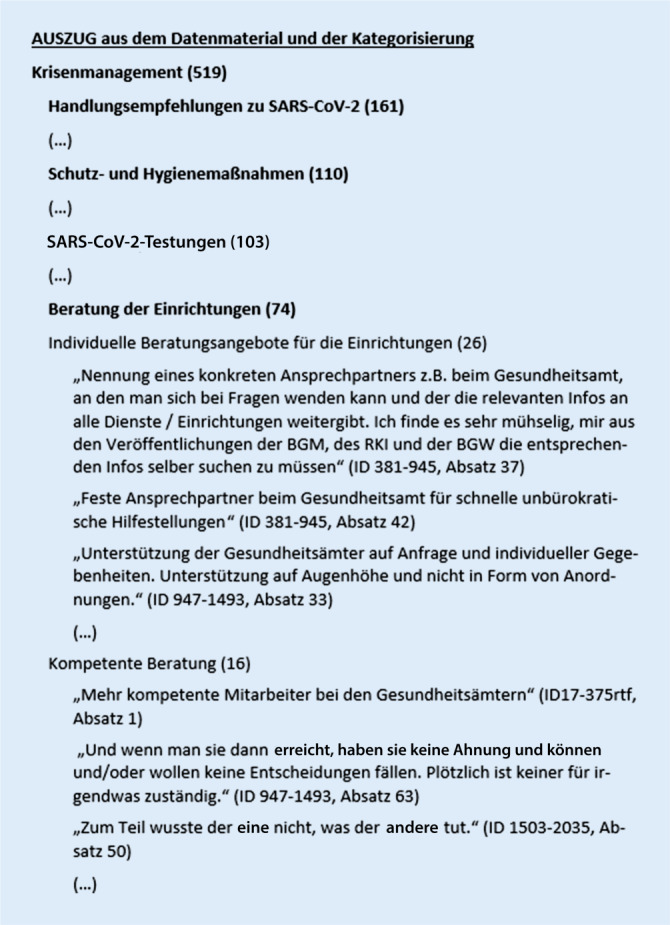

Zusammengefasst in der Unterkategorie *Beratung der Einrichtungen*, benannten die Befragten einrichtungsindividuelle Beratungsbedarfe (*n* = 26) (Abb. 2). Die Einrichtungen suchten *„konkrete Ansprechpersonen“*, die beratend zu Verfügung stünden. Darüber hinaus war den Befragten eine *„bessere Erreichbarkeit“* der zuständigen Institutionen für Rückfragen (*n* = 20) wichtig, und sie berichteten Schwierigkeiten, per Telefon oder E‑Mail Kontaktpersonen für Rückfragen zu finden. 22 % der Befragten gaben an, dass entsprechende Kontaktpersonen nicht immer ausreichend qualifiziert erschienen, um mit praxisnahen Problemlösungen in den Einrichtungen weiterhelfen zu können, sodass ein Bedarf an einer fachlich *„kompetenten Beratung“* bestehe (*n* = 16). Dazu äußerten die Befragten einen konkreten Bedarf an *„Beratung zur Umsetzung“* (*n* = 12) der Vorgaben und Empfehlungen in den Einrichtungen.

Zusammengefasst in der Unterkategorie *Pandemieplan* sind die Forderungen der Einrichtungen nach einer zentralen Bereitstellung von* „Informations- und Schulungsmaterialien“ *(*n* = 20) etwa für Mitarbeiterschulungen oder Angehörigeninformationen. Zur Zeit der Befragung erarbeiteten sich die Einrichtungen diese selbst, was als aufwendig empfunden wurde. Zudem solle ein zentraler *„Pandemieplan“* entwickelt und bereitgestellt werden (*n* = 17), der auf eine Pandemie vorbereite und im Pandemiefall verbindliche Versorgungsprozesse beschreibt und regelt.

In der Unterkategorie *Öffentlichkeitsarbeit* wurden die Wünsche der Befragten nach *„positiver oder neutralen Berichterstattung“ *der Pflege (*n* = 9) zusammengefasst. Aus Sicht der Befragten wurden in der Presse häufig alarmierende negative Szenarien beschrieben, die nicht die tägliche Praxis der Pflege widerspiegeln und ein verzerrtes, negatives Bild vermitteln würden. Die Befragten wünschten sich, *„frühzeitig“* zu zentralen Regelungen informiert zu werden sowie einen Verzicht auf allgemeine *„Panikmache“ *in den Medien (*n* = 8).

### Bedarfe der Pflege

Die Kategorie *Bedarfe der Pflegenden* umfasst insgesamt 158 Textstellen. An erster Stelle benannten die Befragten den *Bedarf an einer „besseren Vergütung“* in der Pflege (*n* = 35), was etwa durch *„Steuerentlastungen“* möglich sei. An zweiter Stelle wurde auf die Notwendigkeit einer „*ausreichenden Personalausstattung“* (*n* = 33) hingewiesen. Die Befragten sahen die Personalausstattung bereits vor der Pandemie als zu gering an. Durch höhere Aufwände im Zusammenhang mit erhöhten Hygieneschutzmaßnahmen und reduzierten Personalkapazitäten in der Pandemie verschärfe sich der Personalbedarf. Im Weiteren forderten die Befragten eine stärkere „*Anerkennung“ und „Wertschätzung“ *der Pflegenden (*n* = 22) in der Langzeitpflege als auch die Wertschätzung weiterer Beschäftigter der Altenhilfe wie Betreuungskräften und Servicepersonal (*n* = 14). Die „*Corona-Prämie“* (*n* = 21) wurde von den Befragten insofern kritisch gesehen, als dass die generelle Forderung nach einer langfristig besseren Vergütung dadurch unberücksichtigt bliebe. Ein Teil der Befragten war der Ansicht, dass die Prämienzahlung auf *„alle“* Beschäftigten in den Pflegeeinrichtungen ausgeweitet werden solle, nicht nur auf die Gruppe der Pflegenden. Im Zusammenhang mit der COVID-19-Pandemie wiesen die Teilnehmenden auf einen deutlich höheren und noch zu vergütenden „*Beratungsaufwand“* (*n* = 10) hin. Dieser entstünde einerseits aus einem erhöhten Informationsbedarf der Angehörigen und andererseits sowohl aus dem Besuchsverbot sowie der fehlenden Unterstützung der Angehörigen in den Pflegeheimen bei der Betreuung der Pflegebedürftigen. An letzter Stelle wurde ein Bedarf nach „*Supervision für Pflegende“* während der Pandemie (*n* = 9) benannt.

## Diskussion

Die Ergebnisse dieser Studie berichten die Rückmeldungen von Einrichtungsleitungen, Pflegedienstleitungen oder Qualitätsbeauftragten stationärer und ambulanter Langzeitpflegeeinrichtungen, die an einer Onlinebefragung zu den Auswirkungen und Folgen der COVID-19-Pandemie teilnahmen. Die Datenauswertung der Sektoren stationäre und ambulante Langzeitpflege erfolgte zunächst getrennt. Abgesehen von Einzelaussagen in den Freitextangaben ließen sich keine eindeutigen unterschiedlichen Bedarfe zwischen den Versorgungsbereichen darstellen, und die Ergebnisse wurden so gemeinsam berichtet. Dies deutet auf vergleichbare Bedarfe der Befragten während der Bewältigung der Pandemie in beiden Versorgungsformen hin.

Da es sich um eine Gelegenheitsstichprobe handelt, die in ihren Strukturmerkmalen leicht von den Einrichtungsmerkmalen der Pflegestatistik abweicht, repräsentieren die Daten nicht alle Langzeitpflegeeinrichtungen, geben aber einen zentralen Einblick in die Bedarfe deutscher Pflegeeinrichtungen.

Die Befragten beschrieben im Erhebungszeitraum einen hohen Bedarf an Schutzmaterialien und Desinfektionsmittel. Ein hoher Anteil der an COVID-19 Verstorbenen entfällt in Deutschland auf Pflegeheimbewohner*innen; jeder 15. Pflegedienst gab mindestens einen COVID-19-Todesfall an [4, 11, 15]. Vor diesem Hintergrund ist unklar, warum in den Pflegeeinrichtungen Ende Mai bzw. Anfang Juni immer noch Engpässe bei der Ausstattung mit Schutzausrüstungen bestanden. Gerade die Langzeitpflege sollte deshalb seitens der Gesundheitspolitik eine höhere Aufmerksamkeit und Unterstützung erfahren, damit die vulnerable Gruppe der Pflegebedürftigen stärker vor der Pandemie und deren Auswirkungen geschützt wird und eine professionelle Versorgung der Pflegebedürftigen sichergestellt ist.

Bei den Maßnahmen zum Schutz der Pflegebedürftigen vor einer SARS-CoV-2-Infektion sollten auch die Arbeitsbelastungen der Pflegenden nicht unberücksichtigt bleiben, denn die schon vor der Pandemie bestehenden hohen physischen und psychischen Arbeitsbelastungen verschärfen sich in der besonderen Situation der Pandemie [6]. Bleiben die hohen Arbeitsbelastungen der Pflegenden bestehen oder steigen weiter an, ist von einer Zunahme von Personalausfällen bei den Beschäftigten auszugehen. Fraglich ist dann, in welchem Ausmaß und wie lange die erforderlichen Schutzmaßnahmen zur Vermeidung einer SARS-CoV-2-Infektion durch Pflegende eingehalten werden, und ob infolge von Personalausfällen eine Eindämmung des SARS-CoV-2-Infektionsgeschehens gefährdet ist. Dass der Gesetzgeber festlegt, dass in der Zeit der Pandemie keine Einleitung von Vergütungskürzungsverfahren nach § 115 Absatz 3 Satz 1 bei Unterschreitungen der vereinbarten Personalausstattung durch die Pflegekassen erfolgen soll, ist angesichts der nunmehr noch angespannteren Personalsituation eine Unterstützung [2], doch weitere Maßnahmen zur Unterstützung der Einrichtungen und zum Schutz der Pflegebedürftigen müssen folgen.

Die Befragten benannten eindeutig externe Beratungsbedarfe der Pflegeeinrichtungen zur Umsetzung von Empfehlungen und Vorgaben zum Infektionsschutz. Zur Entlastung der Langzeitpflege wurde seitens des Gesetzgebers die Qualitätsprüfungen nach § 114 bis einschließlich 30.09.2020 ausgesetzt. Diese bei den medizinischen Diensten der Krankenkassen und dem Prüfdienst des Verbandes der privaten Krankenversicherung e. V. freigewordenen Ressourcen könnten etwa für Beratungsleistungen eingesetzt werden.

Neben der Unterstützung und Entlastung der Pflegeeinrichtungen durch den Gesetzgeber besteht weiterer Forschungsbedarf. Insbesondere sind Erkenntnisse dazu von Interesse, wie Einrichtungen mit einer geringen Anzahl an SARS-CoV-2-Infektionen die Ausbreitung des Virus in ihrer Einrichtung verhinderten und inwieweit sich diese Erkenntnisse auch auf andere Einrichtungen übertragen lassen. Weiter wären die Auswirkungen der Pandemie auf die Lebensqualität und die Versorgungssituation in der Langzeitpflege aus Sicht der Pflegebedürftigen und deren Angehörigen von zentralem Interesse. Insbesondere sind hier Auswirkungen der Präventions- und Hygienemaßnahmen sowie der reduzierten Kontaktmöglichkeiten auf die Kommunikation zwischen Pflegebedürftigen, Pflegenden und Angehörigen, die soziale Teilhabe der Pflegebedürftigen und das physische und psychische Belastungserleben von Pflegebedürftigen und deren Angehörigen zu nennen.

## Limitationen

Die Studie unterliegt Einschränkungen, die bei der Bewertung der Generalisierbarkeit der Ergebnisse zu berücksichtigen sind und sich besonders durch das Vorgehen der Bildung einer Gelegenheitsstichprobe ergeben. Im Vergleich mit den in der Pflegestatistik berichteten Zahlen fällt auf, dass sich tendenziell eher größere Einrichtungen sowie ein höherer Anteil von Einrichtungen in öffentlicher Trägerschaft beteiligten. Die Bedarfe v. a. kleinerer Einrichtungen sind in den Ergebnissen evtl. eingeschränkt abgebildet. Da die Teilnahmemotivation der Befragten weitgehend verschlossen bleibt, ist die Frage nach einer Positiv- oder Negativselektion des Samples zu diskutieren. Eine Unterscheidung der Freitextantworten nach Ausmaß der direkten Betroffenheit einzelner Einrichtungen erfolgte nicht. Jedoch lassen die Formulierungen der Freitextantworten den Schluss zu, dass sich vornehmlich Personen beteiligten, die dem Thema eine hohe Relevanz generell durch die für die Einrichtungen spürbaren Einschränkungen und Herausforderungen zuwiesen. Ob sich Einrichtungen, die besonders stark durch SARS-CoV-2-Infektionen bei Mitarbeitenden oder Pflegebedürftigen betroffen waren, systematisch geringer an der Befragung beteiligten (und somit deren Perspektive unterrepräsentiert ist), bleibt unbekannt. Ebenso spiegeln die Ergebnisse die Sichtweise von Personen mit besonderer Führungs- und Qualitätsverantwortung wieder. Die Bedarfslagen der Mitarbeitenden in der direkten pflegerischen Versorgung werden durch die Studie nicht explizit. Obgleich nicht auszuschließen ist, dass Individualinteressen Einfluss auf das Antwortverhalten ausüben können, ist jedoch anzunehmen, dass die Teilnehmenden ihre Antworten unter Einbezug ihrer alltäglichen Beobachtung der Herausforderungen ihrer Mitarbeitenden formulierten. Mit der Dynamik und den steigenden Erkenntnissen in der Pandemie wurden auch die Empfehlungen zum Umgang SARS-CoV‑2 in den Pflegeeinrichtungen seitens des RKI angepasst. Die hier dargestellten Ergebnisse spiegeln die Situation in den Pflegeeinrichtungen lediglich bis Mitte Mai wieder.

## Schlussfolgerungen

Um die vulnerable Gruppe der Pflegebedürftigen und die Mitarbeitenden von Pflegeeinrichtungen in der aktuellen Situation vor SARS-CoV-2-Infektionen zu schützen, ergeben sich aus den Ergebnissen v. a. Implikationen für politische Entscheidungsträger, die aus den aktuellen Erfahrungen lernen und entsprechende Maßnahmen anbahnen können.

- Begründet mit für sie widersprüchlichen Aussagen der offiziellen Anweisungen, erwarten die Einrichtungen im Pandemiefall bundeseinheitliche und dem jeweiligen Setting angepasste Vorgaben und Empfehlungen. Diese sollten für die Einrichtungen eindeutig, verständlich, praxisnah, umsetzbar sowie handlungsleitend sein und den Rahmenbedingungen des jeweiligen Versorgungsettings klar angepasst sein.
- Aus Sicht der Praxis ist es wichtig, dass Pflegepraxis, -management und -wissenschaft bei der Entwicklung von Pandemieplänen einbezogen werden, damit die Inhalte zielgerichtet an die Praxis adaptiert und dort auch direkt implementiert werden können.
- Im Zusammenhang mit dem Bedarf an der Entwicklung von Pandemieplänen wurde die Bereitstellung zentraler Informationsmaterialen, etwa für die Schulung von Mitarbeitenden und Angehörigen, betont. Bisher ist unklar, warum zentral erstellbare Materialien in der arbeitsintensiven Zeit einer Pandemie von Einrichtungen selbst entwickelt werden und hierfür die – häufig knappen –Ressourcen der Pflegeeinrichtungen aufgebracht werden müssen.
- Es sollten dringend Testkapazitäten und Prozesse ausgebaut werden, mit denen Regeltestungen in den Einrichtungen bei Zunahme der Virusinzidenz in der Region und/oder Cluster-Bildung durchgeführt werden können, um in den Einrichtungen einerseits entsprechend gezielte Maßnahmen vornehmen zu können und andererseits Mitarbeitenden bei negativem Testergebnis zeitnah zu ermöglichen, wieder ihrer Tätigkeit nachgehen können. Eine schnelle Übermittlung der Testergebnisse ist für die Handlungsfähigkeit der Einrichtungen entscheidend.
- Die Einrichtungen müssen mit ausreichend Schutz- und Hygienematerialien versorgt werden, die über zentrale Lager verteilt werden und einer Preiskontrolle unterliegen – nicht nur, aber gerade, im Pandemiefall.
- Beratende Kapazitäten in den Gesundheitsämtern sind auszubauen, um eine verlässliche Erreichbarkeit für Pflegeeinrichtungen und eine qualifizierte, einrichtungsindividuelle und praxisnahe Beratung zur Umsetzung pandemiespezifischer Vorgaben in den Einrichtungen zu fördern.

Über diese kurzfristig zu empfehlenden Maßnahmen lassen sich aus den Daten auch längerfristige Empfehlungen ableiten:

- Der Pflegeberuf sollte eine stärkere Anerkennung und Wertschätzung in der Gesellschaft erfahren. Die Arbeitsbedingungen sollten insbesondere durch eine Verbesserung der Personalausstattung optimiert und die Vergütung der Pflegenden flächendeckend dem Tariflohn angepasst werden.
- Weitere Forschung dazu, wie Einrichtungen effektiv eine Infektion mit dem SARS-CoV‑2 und dessen Ausbreitung verhindern können und welche Bedarfe der Pflegebedürftigen und deren Angehörigen während der Pandemie zu berücksichtigen sind, wird benötigt.

## Fazit für die Praxis

Pflegeeinrichtungen benötigen:

- einheitliche und den Settings angepasste Handlungsempfehlungen zu SARS-CoV‑2,
- ausreichende Schutz- und Hygienematerialien sowie Reihentestungen bei Verdachtsfällen in den Einrichtungen und
- Anlaufstellen zur Beratung bei der Umsetzung der geforderten Schutzmaßnahmen.

Pflegende benötigen:

- eine angemessene Vergütung und Arbeitsentlastung durch angemessene Personalausstattung in den Einrichtungen und
- eine höhere Wertschätzung und Anerkennung der Profession Pflege sowohl während der Pandemie als auch unabhängig von dieser.
